# Editorial: Regulatory pathways in immune microenvironments for tissue development and repair

**DOI:** 10.3389/fcell.2026.1946515

**Published:** 2026-08-27

**Authors:** ChengCheng Yin, Yulan Wang

**Affiliations:** 1 Hospital of Stomatology, Jilin University, Changchun, China; 2 Jilin Provincial Key Laboratory of Oral and Craniofacial Diseases and Tissue Reconstruction, Changchun, China; 3 University of Base, Basel, Switzerland

**Keywords:** cell, immune, mechanism, modulation, regeneration

The successful completion of this Research Topic highlights the increasing recognition that tissue development and repair are not governed solely by intrinsic cellular programs, but are profoundly modulated by the immune microenvironment. Collectively, the four accepted contributions advance our understanding of how immune regulation, biomaterial design, and cellular interactions converge to modulate regeneration across multiple tissues types, including bone, periodontal structures, and the heart.

A central theme arising from this collection is the shift from passive to active modulation of the immune microenvironment. Traditional regenerative strategies have typically focused on the direct stimulation of target cells, such as osteoblasts, while neglecting the dynamic crosstalk between immune cells and tissue-specific progenitors. As demonstrated in these studies, however, immune cells are not mere bystanders but key orchestrators of regeneration, exerting pivotal effects on inflammation, fibrosis, and tissue remodeling.

The work by Liu et al., titled “Precise Copper Ion Release and Recovery in Polycaprolactone Nanofiber Scaffold: An Antibacterial and Osteogenic Synergistic Strategy for Guided Bone Regeneration,” illustrates how biomaterial engineering can precisely regulate immune-relevant factors. By integrating polydopamine (PDA) and copper ions into polycaprolactone (PCL) nanofiber scaffolds, the study developed a system capable of controlled ion release and recovery. This design is particularly notable in that it balances antibacterial activity with osteogenic promotion-two processes often in tension due to cytotoxicity concerns. The ability to maintain copper ion concentrations within an optimal therapeutic window underscores the importance of temporal and environmental responsiveness in biomaterial design. Such strategies are consistent with the broader concept of tailoring the immune microenvironment to promote regeneration while minimizing adverse effects.

Complementing this work, Zhao and colleagues, in their review “From ‘Immune Silence’ to ‘Immune Dialogue’: Modification Strategies for Bone Substitutes Based on Bone Immunoregulatory Characteristics,” present a conceptual and systematic examination of biomaterials from an osteoimmunology perspective. The authors propose a paradigm shift from “immune silence” to “immune dialogue,” emphasizing that successful bone substitutes must actively engage with the host immune system. Through the modulation of macrophage polarization, control of bioactive ion release, and incorporation of responsive systems, next-generation materials can dynamically adapt to the local microenvironment. This perspective not only challenges conventional design principles but also establishes a framework for integration of immune-metabolic regulation into clinical biomaterials.

Beyond bone tissue, the importance of the immune microenvironment is further demonstrated in periodontal regeneration. Addressing the long-standing challenge of cementum regeneration, Wang et al. provide an insightful review titled “Cementum regeneration strategies: insights from development and periodontal microenvironment,” which highlights the complexity of the periodontal niche, including host cells, microbial communities, and metabolic factors. This review synthesizes current knowledge regarding cementum development, with particular emphasis on regulatory mechanisms such as metabolic reprogramming, epigenetic modifications, and immune-stem cell interactions. By linking developmental biology to regenerative strategies, this work provides valuable insights into how microenvironmental cues can be leveraged to overcome the limitations of traditional therapies.

Extending the scope of this Research Topic beyond tissue engineering, the study by Zhao and co-workers, entitled “Integrated Analysis of Cell-in-Cell Related Genes and Immune Microenvironment in Heart Failure,” explores the immune microenvironment in the context of heart failure (HF)—a condition not traditionally linked to regenerative medicine. Through integrated bioinformatics and experimental validation, the study identifies cell-in-cell (CIC)- related genes as key regulators of immune heterogeneity in HF. The classification of HF into immunosuppressive and immune-activated subtypes based on CIC-related gene expression underscores the functional diversity of immune responses in disease progression. Importantly, this work demonstrates that analysis of the immune microenvironment can provide diagnostic value and inform targeted therapeutic strategies, reinforcing the relevance of immune regulation across diverse pathological contexts.

Collectively, these contributions highlight several key advances in the field:

Precision modulation of the immune microenvironment is critical for balancing competing processes, including inflammation, antibacterial defense, and tissue regeneration.

Biomaterials are evolving from passive scaffolds to active regulators, capable of dynamically interacting with immune and stem cell populations.

Cross-disciplinary integration, including bioinformatics, single-cell analysis, and experimental validation, is essential for deciphering complex immune-tissue interactions.

Immune heterogeneity plays a decisive role not only in regeneration but also in disease progression, offering new avenues for diagnosis and therapy.

Despite these advances, several challenges persist. A deeper understanding of spatiotemporal immune dynamics is required to optimize therapeutic interventions. Furthermore, the translation of these findings into clinical applications will necessitate rigorous validation, standardized models, and consideration of patient-specific variability. Future research should also explore the integration of artificial intelligence and systems biology to predict and modulate immune responses in real time.

In conclusion, this Research Topic highlights the transformative potential of targeting immune microenvironments in tissue development and repair. By integrating fundamental biology with innovative engineering approaches, these studies lay the foundation for more effective and personalized regenerative therapies.

